# Where have all the mosquito nets gone? Spatial modelling reveals mosquito net distributions across Tanzania do not target optimal *Anopheles* mosquito habitats

**DOI:** 10.1186/s12936-015-0841-x

**Published:** 2015-08-19

**Authors:** Emily S. Acheson, Andrew A. Plowright, Jeremy T. Kerr

**Affiliations:** Department of Biology, University of Ottawa, Gendron 352, 30 Marie Curie, Ottawa, ON K1N6N5 Canada

**Keywords:** *Anopheles* mosquito, Insecticide-treated nets, ITN, Malaria, Modelling, Niche, Species distribution models, Vector

## Abstract

**Background:**

Malaria remains the deadliest vector-borne disease despite long-term, costly control efforts. The United Republic of Tanzania has implemented countrywide anti-malarial interventions over more than a decade, including national insecticide-treated net (ITN) rollouts and subsequent monitoring. While previous analyses have compared spatial variation in malaria endemicity with ITN distributions, no study has yet compared *Anopheles* habitat suitability to determine proper allocation of ITNs. This study assesses where mosquitoes were most likely to thrive before implementation of large-scale ITN interventions in Tanzania and determine if ITN distributions successfully targeted those areas.

**Methods:**

Using Maxent, a species distribution model was constructed relating anopheline mosquito occurrences for 1999–2003 to high resolution environmental observations. A 2011–2012 layer of mosquito net ownership was created using georeferenced data across Tanzania from the Demographic and Health Surveys. The baseline mosquito habitat suitability was compared to subsequent ITN ownership using (1) the average ITN numbers per house and (2) the proportion of households with ≥1 net to test whether national ITN ownership targets have been met and have tracked malaria risk.

**Results:**

Elevation, land cover, and human population distribution outperformed variants of temperature and Normalized Difference Vegetation Index (NDVI) in anopheline distribution models. The spatial distribution of ITN ownership across Tanzania was near-random spatially (Moran’s I = 0.07). Householders reported owning 2.488 ITNs on average and 93.41 % of households had ≥1 ITN. Mosquito habitat suitability was statistically unrelated to reported ITN ownership and very weakly to the proportion of households with ≥1 ITN (R^2^ = 0.051). Proportional ITN ownership/household varied relative to mosquito habitat suitability (Levene’s test F = 3.0037). Quantile regression was used to assess trends in ITN ownership among households with the highest and lowest 10 % of ITN ownership. ITN ownership declined significantly toward areas with the highest vector habitat suitability among households with lowest ITN ownership (t = −3.38). In areas with lowest habitat suitability, ITN ownership was consistently higher.

**Conclusions:**

Insecticide-treated net ownership is critical for malaria control. While Tanzania-wide efforts to distribute ITNs has reduced malaria impacts, gaps and variance in ITN ownership are unexpectedly large in areas where malaria risk is highest. Supplemental ITN distributions targeting prime *Anopheles* habitats are likely to have disproportionate human health benefits.

**Electronic supplementary material:**

The online version of this article (doi:10.1186/s12936-015-0841-x) contains supplementary material, which is available to authorized users.

## Background

Malaria remains the most deadly of all vector-borne diseases, infecting an estimated 200 million people and causing over 550,000 deaths globally every year [1]. Approximately 90 % of all malaria deaths occur in Africa, where an estimated 78 % of deaths are in children under the age of five [1]. Malaria parasites are transmitted exclusively by *Anopheles* mosquitoes [2], spurring decades of research not only on the parasite, but also on its widespread vector. The spatial variation in vector-borne disease distributions such as malaria can likely be attributed to variations in environmental conditions (e.g. land cover, temperature, precipitation) and human population distribution that disease vectors depend on for survival [3]. Ecological niche models (ENMs) correlate these environmental factors with georeferenced occurrences of *Anopheles* mosquitoes to improve detection of spatial and temporal variation in biophysical determinants of these malaria vectors, often at a countrywide or even continent-wide scale [4–8]. Although these modelling methods are rapidly advancing, they have yet to incorporate anti-malarial intervention data, though such approaches have been suggested and encouraged [2]. Anti-malarial controls are not only time-intensive but also financially burdensome, with $5.1 billion US planned but $2.7 billion US expended in 2013 [1]. The synthesis of species distribution modelling (or ecological niche modelling) and large-scale mosquito control methods can offer a new perspective on the potential effects of anti-malarial controls [4].

Insecticide-treated nets (ITNs) are an excellent example of such vector control methods working on a large scale. The percent reduction in malaria deaths in children under the age of five was estimated in 43 sub-Saharan African countries from 2001 to 2010, compared to a baseline in 2000. Some countries reported no reduction by ITN scale-ups, while others reported up to a 26 % decrease in malaria deaths in children under the age of five [9]. In 2014, a record number of ITNs were distributed to malaria-endemic African countries, with the total number of ITNs delivered to Sub-Saharan Africa reaching 427 million [1]. Within the United Republic of Tanzania alone (hereafter Tanzania), an extensive ITN national plan has been implemented over the past 25 years, supported by the Global Fund to Fight AIDS, Tuberculosis and Malaria (GFATM) and the USA President’s Malaria Initiative [10]. Since 2004, the Tanzania National Voucher Scheme has provided subsidized ITNs to pregnant women during antenatal visits [11]. Between 2009 and 2010, a mass campaign distributed 8.7 million ITNs across the country, which were free to families with children under the age of five [12]. Despite increases in coverage, analyses of ITN scale-up impacts [9] found only a 3 % reduction in malaria deaths in Tanzanian children under the age of five from 2001 to 2010. In 2011, a Universal Coverage Campaign distributed 17.6 million ITNs nationally with the goal to increase global use in the general population to 80 % [10]. In addition, the GFATM set a national target for Tanzania to increase the proportion of households with at least one ITN to 90 % by 2013 [13].

Numerous analyses have been conducted to determine the effects of ITN use on malaria endemicity [14–17] and on mosquito populations at local scales [18–20], but none have yet compared ITN coverage with mosquito habitat suitability to determine if coverage has been optimally allocated. Environments that favour *Anopheles* mosquito survival and reproduction, as well as more densely populated areas that facilitate access to hosts and accelerate malaria parasite transmission, will have higher malaria risk. Given mass ITN rollouts, is ITN ownership across Tanzania meeting targets in areas with high mosquito habitat suitability and increased malaria risk? Furthermore, if ITN distributions were optimized to target the most at-risk groups, areas with greater mosquito habitat suitability would be expected to have both greater averages and lower variances in ITN ownership rates (because coverage rates should be consistently high in malaria-endemic areas). Yet, ITN ownership relative to vector suitability remains uncertain even in regions, such as Tanzania, where national household surveys exist to permit such assessments.

Here, a species distribution model is created at 1-km resolution of *Anopheles* mosquitoes across Tanzania in 2001 (before large-scale ITN distributions) and is compared with countrywide ITN ownership by 2012 (number of ITNs owned per house and proportion of houses with at least one ITN) to assess where mosquitoes were most likely to thrive and whether ITN rollouts ensured coverage of such areas. This study uses the largest collection known of *Anopheles* mosquito occurrence records in Tanzania available for this time period, including 400 published sources, private data collections and online IR Mapper and the Malaria Atlas Project databases. This study relates species distribution models for *Anopheles* mosquitoes to ITN ownership and may serve as a template for integrating *Anopheles* mosquito distributions and disease risk with anti-malaria interventions.

## Methods

### Study area

**Fig. 1:**
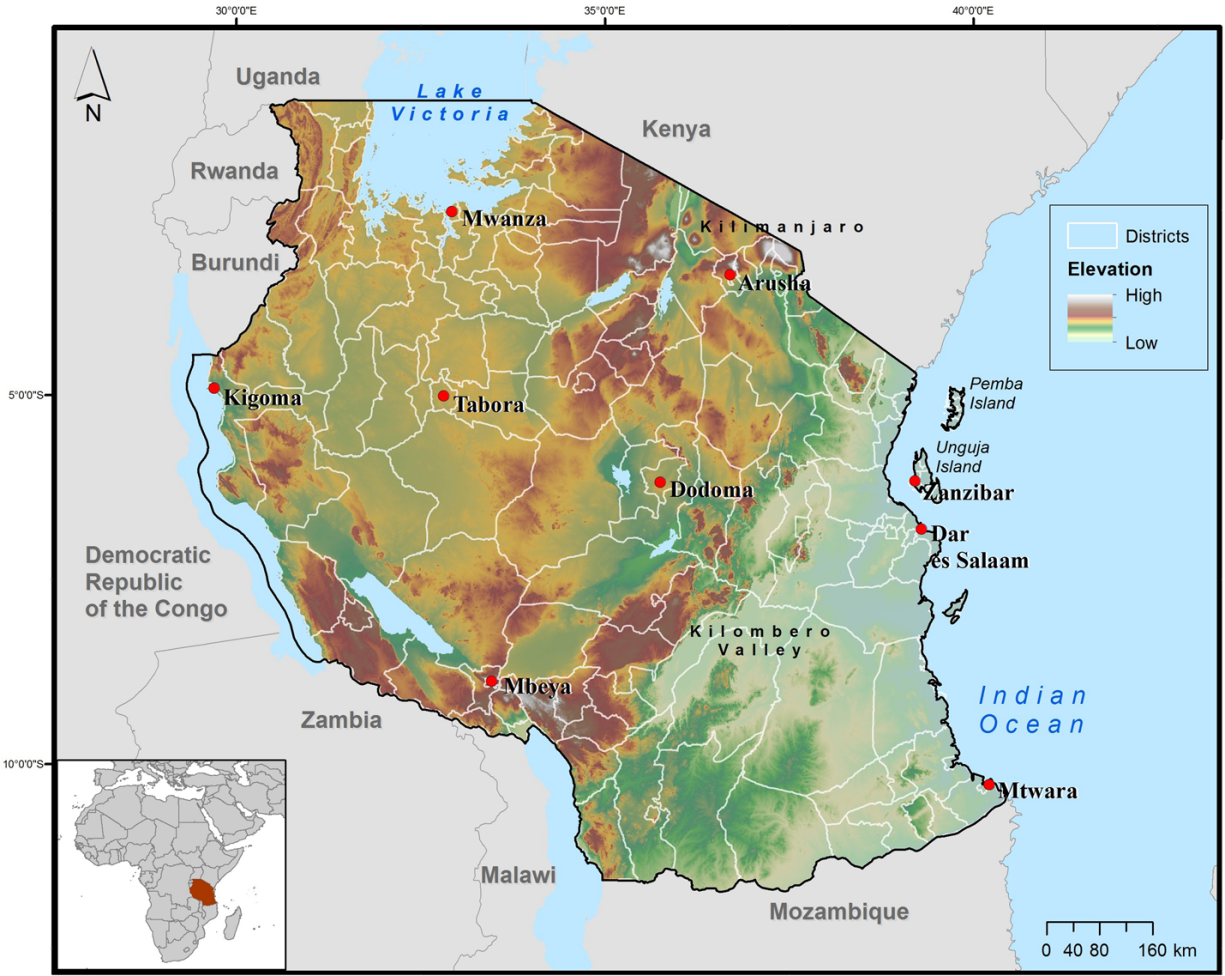
United Republic of Tanzania, detailed by districts and elevation. The map shows the 148 Demographic and Health Surveys “admin 2” district boundaries, as well as elevation, several major cities, and bordering countries. The *inset* map details Tanzania’s location within Africa

The study area included all of Tanzania, including the large islands in the Indian Ocean (Fig. 1). Tanzania occupies 886,100 km^2^ [21] and its population exceeded 45 million people by 2012 [22]. Tanzania remains one of the poorest countries in the world, with a per capita average yearly income of $630 US as of 2013 [22]. Of all vector-borne diseases in the country, malaria still causes the highest morbidity and mortality rates [23]. Over 85 % of cases are caused by *Plasmodium falciparum*, the most dangerous malaria parasite [24].

The country has a tropical climate with wet and dry seasons that vary due to its topography. The northern and eastern regions experience two wet seasons, with short rains from October to December and long rains from March to May. The western, central, and southern regions mainly have one wet season that lasts from October to April or May. About 80 % of the population lives in rural arid regions [25] at very low population densities, with the remaining population in high-density urban centers, such as Dar es Salaam, which approaches 3133 people/km^2^ [26]. Mosquito net coverage following massive ITN rollouts is not related to wealth but prioritizes pregnant women and families with children under the age of five [10].

### Entomological records

The main malaria vectors in Tanzania are *Anopheles arabiensis*, *Anopheles funestus*, and *Anopheles gambiae* s.s. [27]. Over 400 published literature sources were reviewed for georeferenced occurrences of *Anopheles* mosquitoes in Tanzania. An observation for *Anopheles* species was recorded if collection localities were specified for any month and year of collection between 1934 and 2014. These data were supplemented by unpublished data collections (personal comm. Dr. Maureen Coetzee) and online database IR Mapper [28] and the Malaria Atlas Project database [29]. Each coordinate was verified using Google Earth (Version 7.1.2.2019, Google Inc.).

When complete, the database comprised 203 unique georeferenced observations of *An. gambiae* s.l. (35.5 %), *An. arabiensis* (32.4 %), *An. funestus* s.l. (16.2 %), and *An. gambiae* s.s. (15.9 %) across all years. Identification of mosquitoes within the *An. gambiae* s.l. complex relied on polymerase chain reaction or cytogenetics. The database details species, month and year of collection, citation source, village of collection, and trapping method.

### Niche model

Maxent software [30], v.3.3.3k, was used to predict the relative habitat suitability of *Anopheles* mosquitoes across Tanzania. Maxent combines presence-only species occurrence records with environmental data to create a model that predicts areas of relative habitat suitability for a given species. Maxent uses a background dataset that consists of pseudoabsences (i.e. a random sample of non-occurrences) instead of true recorded absences. The output estimates relative habitat suitability and not direct occurrence probability since areas where the species was not recorded are considered less suitable relative to where the species was actually recorded. Maxent niche models with high training accuracy have been constructed for arthropod vectors, including mosquitoes [4, 31–33], ticks [34–36], tsetse flies [37–39] and sandflies [40–42]. Despite Maxent’s tendency to be more conservative than other machine-learning techniques [43], it is one of the most reliable species distribution modelling methods, even with small numbers of species observations [44, 45]. However, internal training and testing accuracies evaluate the model’s fit to existing observations and external tests against independent measurements or temporal environmental changes provide stronger tests of model performance [46].

**Fig. 2:**
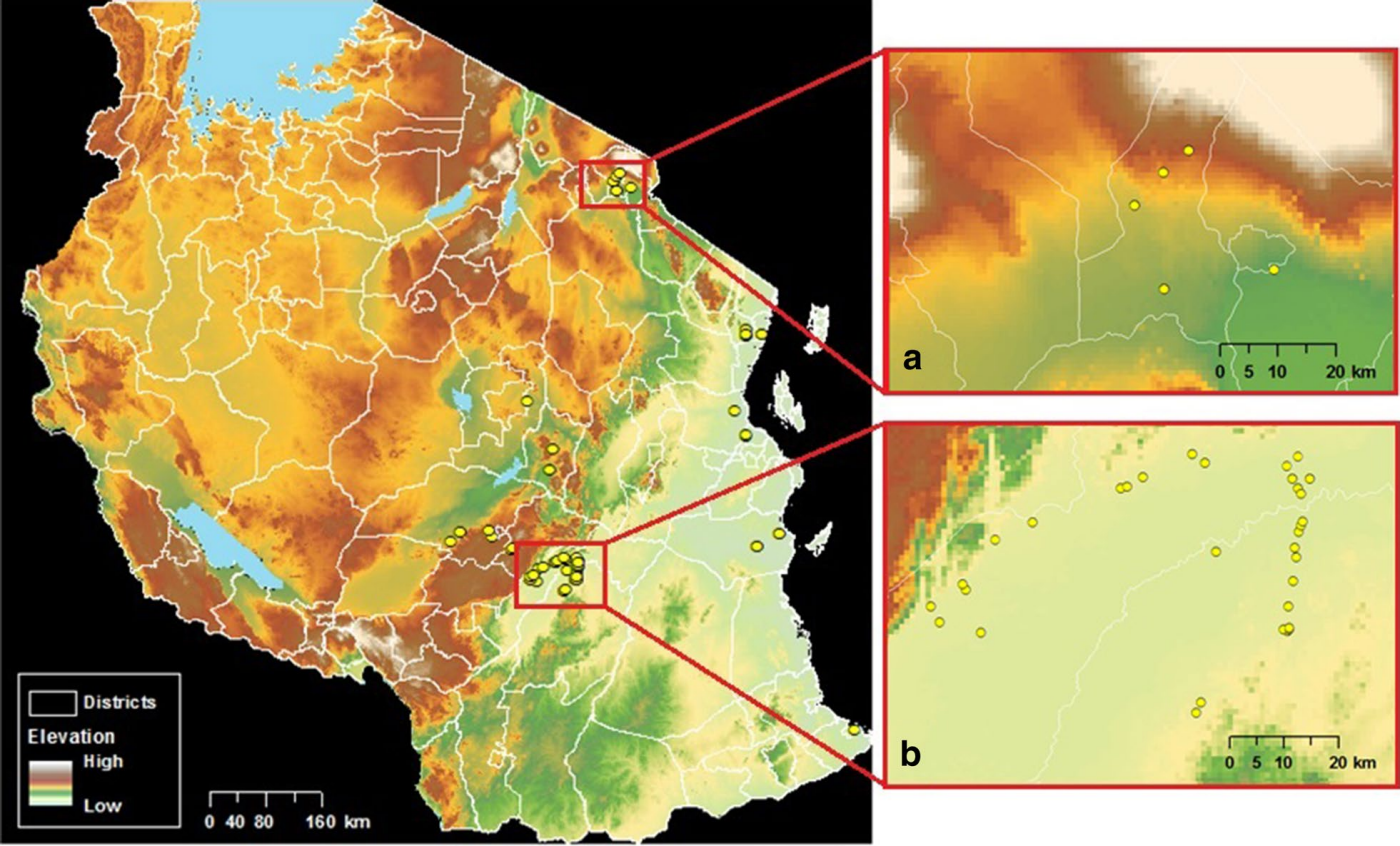
Map of 56 *Anopheles* occurrence points used in Maxent. *Insets* show clustering of biased sampled points around **a** Mount Kilimanjaro and **b** Kilombero Valley

Since large-scale mosquito net distributions commenced in 2004, the niche model building was narrowed to the *Anopheles* coordinates collected between 1999 and 2003. Many historical records for anopheline mosquitoes in the 1999–2003 period do not distinguish species within the *An. gambiae* s.l. complex (e.g. only three geographically-unique points were recorded as *An. gambiae* s.s. compared to 54 recorded as *An. gambiae* s.l.). All recorded *Anopheles* species were combined to build the Maxent model. The focus of this study includes any of these malaria vectors, despite niche differences among species, relative to mosquito net ownership. All eight species within the *An. gambiae* s.l. complex can transmit malaria parasites except *Anopheles amharicus*, which does not occur in Tanzania, and *Anopheles quadriannulatus*, which has not been recorded in the country since 1968 [47]. The absence of spatially detailed data sufficient for these models prevented species-by-species evaluation of habitat suitability for the time period relevant to national ITN distribution. In total, 56 occurrence records were thus used to build the Maxent model, substantially exceeding minimum requirements (>5 observations) to produce informative predictions [44] (Fig. 2). Of these records, one was *An. arabiensis*, seven were *An. funestus*, 46 were *An. gambiae* s.l. and two were *An. gambiae s.s*., though more than one species was recorded at each location.

**Fig. 3:**
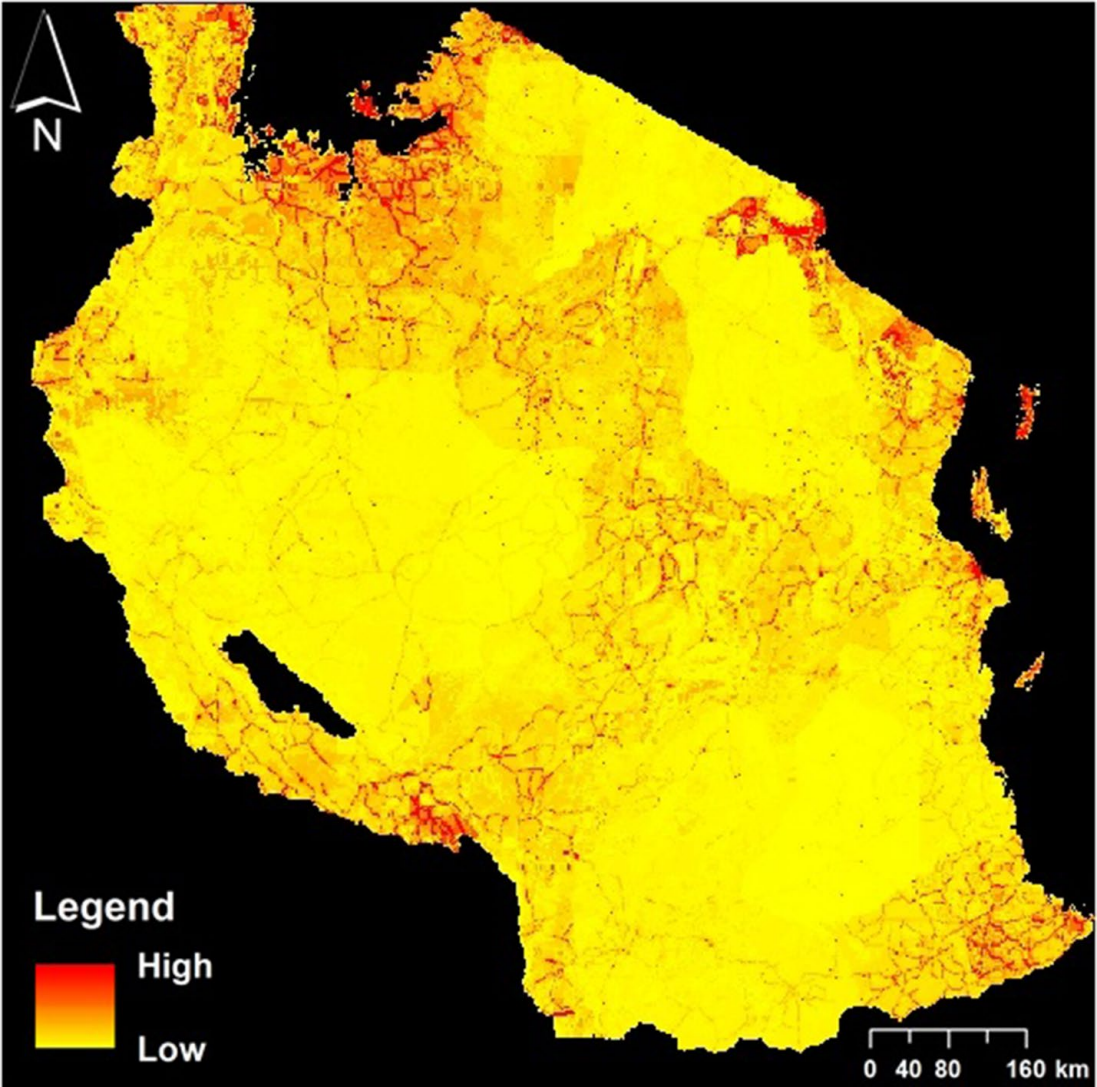
Map of human population distribution from LandScan with pixel size of ~1 km^2^

Multiple models were constructed using elevation, human population (Fig. 3), land cover, and variations of temperature and NDVI (average, minimum, and maximum). For assessment of model accuracy, occurrence records were randomly partitioned into 75 % for model training and 25 % for model testing. Ten replicates were run for each model, using tenfold cross-validation, each with a randomized partitioning of training and testing data, a technique that can reliably test spatial model skill for disease vector distributions [46]. The habitat suitability raster maps were then averaged to determine the relative probability of suitability per grid cell.

The model’s accuracy was determined using a threshold-dependent binomial omission test and a threshold-independent receiver operating characteristic analysis [30]. For the binomial test of omission, a fixed threshold for habitat suitability values of 0.1 was used, which is a threshold commonly set in other studies [4, 43]. For the threshold-independent analysis, model skill was evaluated using area under the receiver operating characteristic (AUC) [4, 48, 49]. AUC values approaching 1 indicate perfect discrimination between suitable and unsuitable areas for the target species, and values of 0.5 indicate performance no better than random. Each variable’s unique and shared contribution to model accuracy was evaluated using jackknife procedures [30]. Each variable’s relative contribution to overall model predictions was then calculated and presented as a percentage.

### Environmental data

The histories of many mosquito-borne diseases, including malaria, indicate that climate is rarely the principal determinant of mosquito distributions in tropical regions and that shorter-term consequences of human activities often exert greater effects [50, 51]. Relevant climatic factors include land surface temperature and precipitation [4, 43]. Models incorporating satellite-based land cover and/or topography as indicators of mosquito habitats can pinpoint local variations in malaria risk [52]. Human population density is a critical determinant of relative malaria risk because of density-dependent transmission of parasites among individuals in the presence of anthropophilic *Anopheles* vectors [43].

Measurements of environmental conditions were used at 500–1000 m resolution across Tanzania derived using the Moderate Resolution Imaging Spectroradiometer (MODIS) sensor on the Terra satellite. All 8-day Land Surface Temperature composites (LST; MOD11A2, 1000 m resolution), 12 monthly Normalized Difference Vegetation Index composites (NDVI; MOD11A3, 1000 m), and land cover observations (MCD12Q1, 500 m) were acquired for 2001. NDVI measures greenness and photosynthetic activity, which detects vegetation differences due to irrigation (such as rice-growing areas). Digital elevation data at 90-m resolution were obtained from the Shuttle Radar Topography Mission (SRTM; [53]) and mosaicked for the terrestrial area of Tanzania. Finally, 2001 human population distribution data were collected from the OakRidges National Laboratory LandScan database at 30 arc second (~1 km) resolution [54] (Fig. 3).

The MODIS data were unprojected from Sinusoidal to geographic coordinate system (GCS WGS 1984 datum) using the MODIS Reprojection Tool (MRT, v.4.0 from US Geological Survey). Human population and elevation layers were transformed to the same geographic coordinate system using ArcGIS v.10.1 (ESRI 2012, Redlands, CA). Some 8-day LST and NDVI mosaics pixels were flagged as missing or of low quality, usually due to atmospheric haze or cloud cover. Data were imported into R v.3.0.2 [55] and the raster brick function (package *raster*) was used to extract mean, minimum and maximum temperature and NDVI, omitting missing and low-quality pixels (see Additional file 1 for more details about the R script used for the raster brick function). Raster bricks for temperature and NDVI were then exported to ArcGIS 10.1 and all environmental layers were clipped to a land mask for Tanzania and converted to ASCII for use in Maxent.

### Mosquito net survey data

This study used 2011–2012 data conducted by the AIDS Indicator Survey (AIS) throughout Tanzania, with permission from the Demographic and Health Surveys (DHS) Programme [56], funded primarily by the US Agency for International Development. AIS fieldwork was conducted between December 2011 and May 2012, implemented by Tanzania’s National Bureau of Statistics. This survey provided data on number of ITNs owned per household, including long-lasting insecticide nets (LLINs). All DHS procedures and questionnaires were reviewed and approved by the ICF International Institutional Review Board. Further details are provided in each survey’s final report.

To protect respondent confidentiality, the georeferenced cluster points (i.e. sample points that represented 17–18 surrounding households) were each displaced by the DHS, with shifts in latitude and longitude under set parameters [57]. Urban clusters were displaced 0–2 km and rural clusters were displaced 0–5 km, with 1 % (or every 100th point) displaced from 0 to 10 km. Shifts in direction and distance were both random. In addition, displacement of the georeferenced clusters was restricted at the district (admin2) level for the 2011–2012 AIS survey (Fig. 1). During the randomized cluster displacement process, clusters could cross localized administrative boundaries (e.g. ward boundaries), but not a DHS region or district boundary.

### Mosquito net ownership layers

**Fig. 4:**
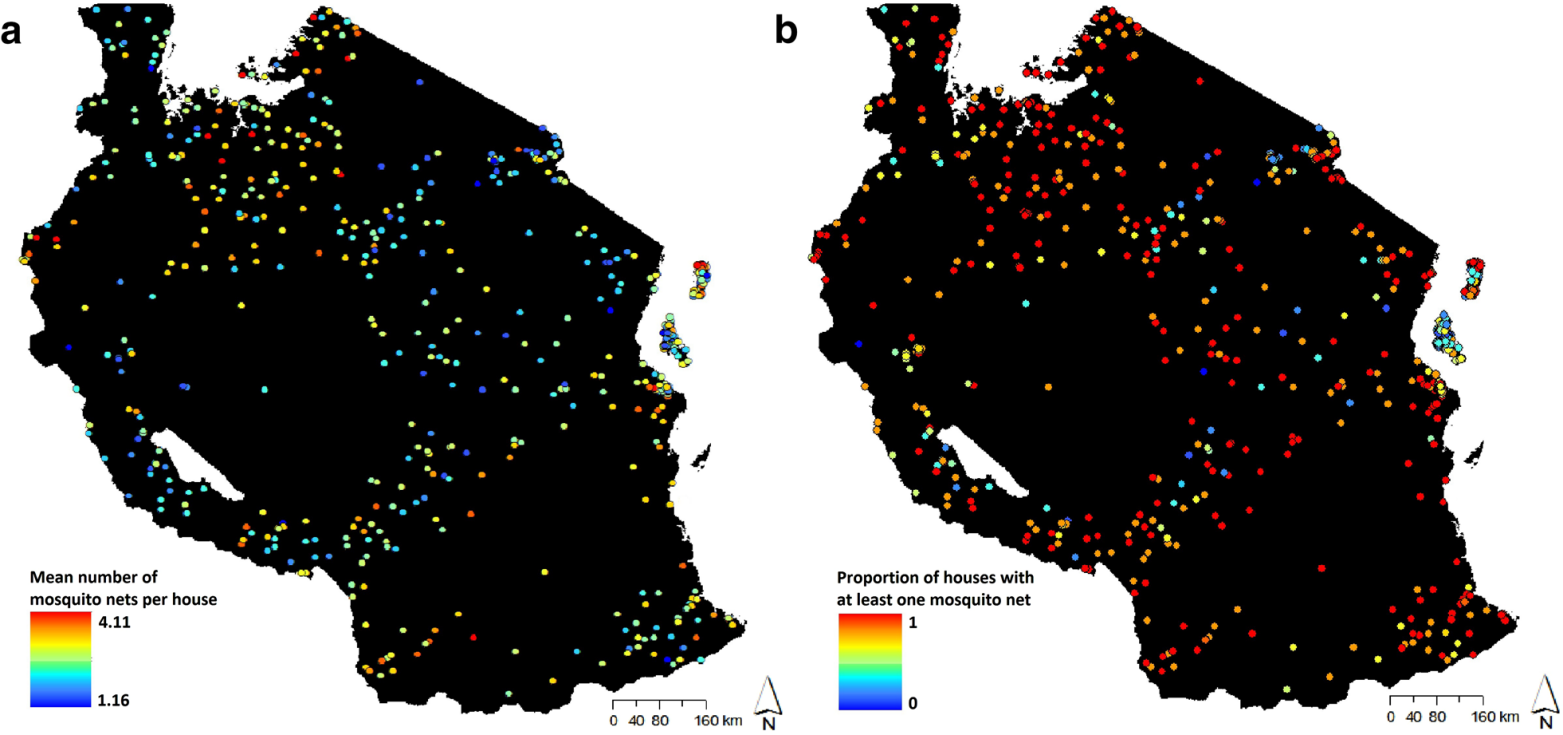
The 573 raw georeferenced cluster points provided by the 2011–2012 DHS survey. Cluster points are *colour coded* to represent spatial variation in **a** average number of mosquito nets used per household or **b** the proportion of households with at least one mosquito net across Tanzania

For the 2011–2012 AIS survey for Tanzania, 10,496 households were selected for sampling, of which 10,040 were successfully sampled and included in the final survey results. The survey’s GPS dataset comprised of 583 georeferenced survey cluster points, where each cluster point represented 17–18 surrounding households. Ten clusters were removed due to missing data. Of the remaining 573 clusters, 440 were in rural locations and 133 were in urban locations. Using this data set, mosquito net ownership was measured in two ways: first, the average number of mosquito nets owned per household and, second, the proportion of houses with ≥1 mosquito net (Fig. 4). While both offer distinct perspectives on mosquito net ownership, the latter is particularly relevant to the stated national target of 90 % of households with at least one mosquito net [13]. For each layer, the resulting calculated value from the surrounding households was assigned to their corresponding cluster coordinate.

Mosquito net ownership patterns were expected to be spatially autocorrelated and be used to estimate mosquito net ownership rates throughout unsurveyed areas of Tanzania. To test this possibility, extrapolations were attempted using kriging and inverse distance weighting techniques in ArcGIS. Predicted mosquito net ownership from extrapolated surfaces were compared to observed mosquito net ownership rates using tenfold cross validation, but model fit was very poor. Mosquito net ownership shows practically no spatial autocorrelation (measured using Moran’s I on cluster points = 0.07, p < 0.05, where Moran’s I scales as the Pearson product-moment correlation between 1 and −1, with 0 indicating no autocorrelation). Further analyses were restricted to point-based measurements.

Buffer zones were created surrounding each survey cluster, with urban clusters having a circular buffer zone with a radius of 2 km and rural clusters having a circular buffer zone with a radius of 5 km. Since cluster points were randomly relocated but did not cross district boundaries, buffer zones that crossed into an adjacent district could be made more spatially precise by clipping along the district boundary. Buffer zones were consequently retained only within the district particular to each survey locality. This processing resulted in a total of 912 mosquito net polygons. The area of the buffer zone was assigned the value attributed to its cluster point for the average number of mosquito nets and the proportion of houses with ≥1 ITN. For overlapping buffer areas, the averaged value of the individual buffers was calculated (see Additional file 2 for the R script used to create this layer).

### Statistical analyses

To relate mosquito habitat suitability to mosquito net ownership, the habitat suitability map created in Maxent and the mosquito net buffer layers created in ArcGIS 10.1 were imported into R statistical software (v.3.0.2, [55]). Since each buffer zone had either a 2- or 5-km radius, and had been clipped depending on proximity to district boundaries, R was used to calculate average habitat suitability for each unique cluster and surrounding buffer (see Additional file 3 for the R script used for this process). Ordinary least squares (OLS) regression models were then constructed to test whether *Anopheles* habitat suitability predicted either average numbers of mosquito nets owned per household or proportions of households with ≥1 mosquito net. For each regression analysis, Levene’s tests were used to test whether variances differed between household clusters that were grouped by deciles according to predicted suitability for anopheline mosquitoes. Residual spatial autocorrelation and the associated Moran’s I were calculated using R. In addition, possible relationships were tested between the two measures of mosquito net ownership as a function of mosquito habitat suitability by performing quantile regression analyses in the 0.1 (the 10 % of household clusters with the lowest predicted suitability for mosquitoes) and 0.9 (the 10 % of household clusters with the highest predicted suitability for mosquitoes) deciles. Quantile regression is a method for providing a more complete view of possible causal relationships in ecological processes aside from the mean of the response variable distribution [58]. Using quantile regression to analyse the highest and lowest 10 % of the data may reveal changes in ITN coverage within these households as mosquito habitat suitability increases that could be undetected by common regression models through the mean of the response variable. If ITN ownership is consistently high, regardless of an area’s predicted suitability for anopheline mosquitoes, no trend is expected (slope = 0) among quantiles. Less favourably, ITN ownership rates could increase toward areas with high suitability for these vectors, suggesting that priority for ITN distribution had been given to localities with the greatest malaria risk. Finally, ITN ownership might decline toward areas with high anopheline suitability.

## Results

### Habitat suitability model

**Fig. 5:**
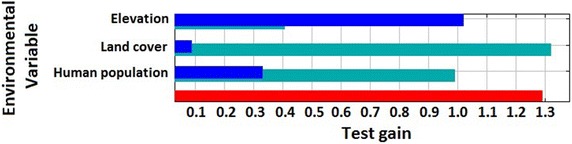
Jackknife plot of testing gain for Maxent niche model building. *Green bars* represent test gain without the specified variable. *Blue bars* represent test gain with only the specified variable. The *red bar* represents the test gain with all variables included

**Fig. 6:**
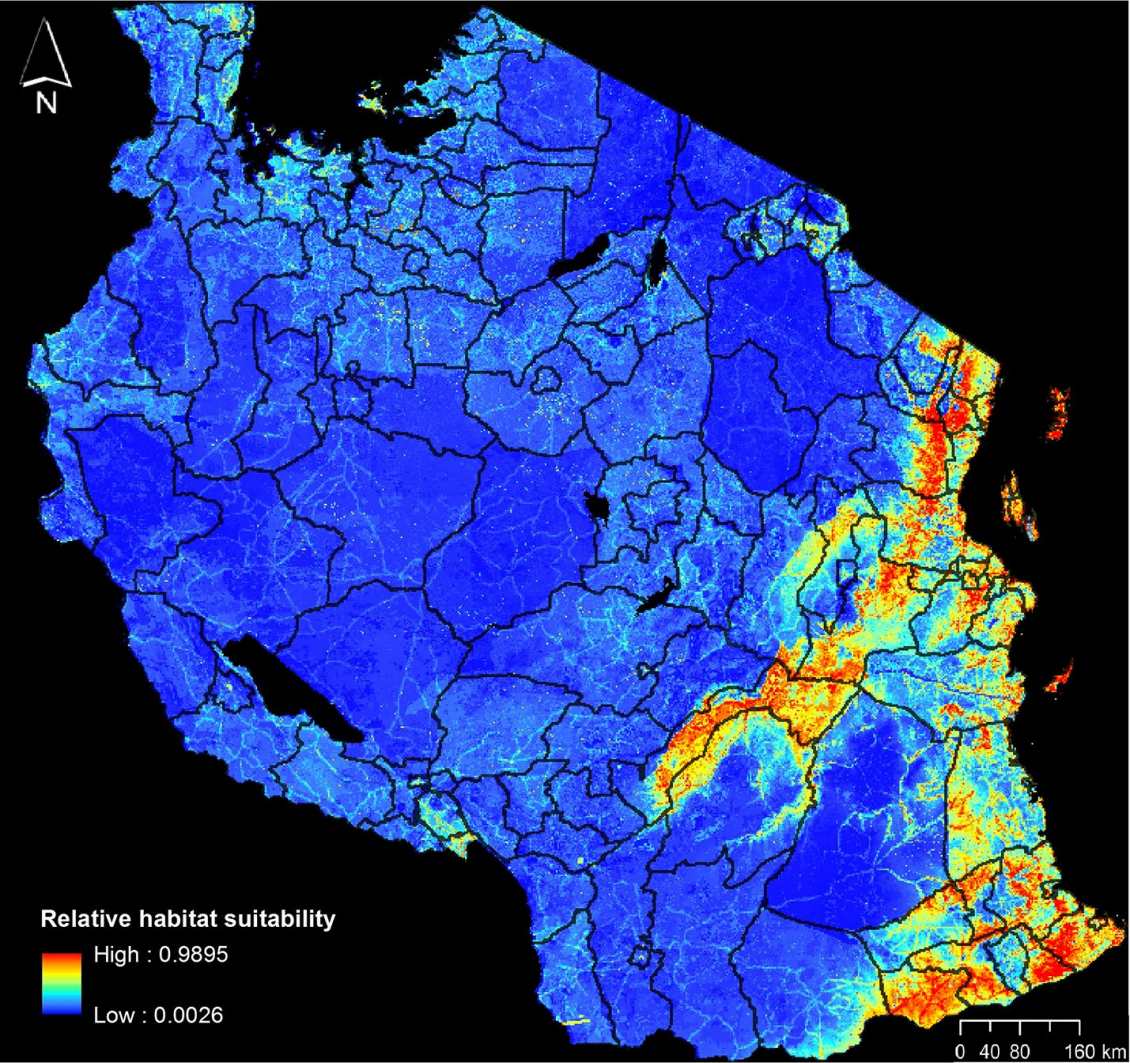
Maxent output of relative *Anopheles* mosquito habitat suitability (n = 56). The habitat suitability map ranges from *blue* to *red*, with *blue* representing lowest predicted relative habitat suitability for *Anopheles* mosquitoes. The suitability map is overlaid with the “admin 2” districts of Tanzania. Predicted relative habitat suitability in many areas are coterminous with the LandScan human population data (e.g. the southeastern polygonal area of low habitat suitability corresponds substantially with the Selous Game Reserve, where human population approaches zero in most areas.)

Elevation, human population distribution and land cover provided stronger predictions of mosquito habitat suitability than any temperature- or NDVI-based measurements (Table 1). Model fits were comparable whether the latter measurements were omitted (AUC = 0.872) or included (AUC = 0.862–0.875). Elevation made the greatest contribution to the model, followed by human population distribution and land cover. Jackknife analysis attributed the maximum training gain to elevation (0.828), which indicates this variable contains the most information that is not present in the other variables (Fig. 5). Concentrated areas of high habitat suitability are found in low elevation areas, mainly along eastern coastlines and extending into Kilombero Valley and south toward Mozambique (Fig. 6). Areas surrounding Lake Victoria and north of Lake Malawi also contain extensive areas with high habitat suitability for anopheline mosquitoes. Dense shrub land and cropland/natural vegetation mosaic land covers were associated with higher mosquito habitat suitability. Internal accuracy of the model through the threshold-dependent binomial omission test was also high, with an omission error rate of 7.14 % with a suitability threshold of 0.1 with the omission error rate remaining below 20 % up to a set threshold of 0.25 (n = 56). Removing elevation from Maxent models degraded model fit substantially (AUC = 0.765), increasing proportional contributions of human population distribution and land cover (65 and 25.1 %, respectively), with minor increases in temperature and NDVI (7.1 and 2.8 %, respectively). The final model excluding temperature and NDVI, but maintaining a high AUC value of 0.872, was retained.

**Table 1:** Contribution of environmental variables to *Anopheles* Maxent model, including and excluding variants of temperature and NDVI

Variable	% Contribution to model	AUC with variable alone	AUC without variable	Model AUC
Model 1				
Elevation	66.2	0.817	0.772	0.869
Human population distribution	28.2	0.727	0.773	
Land cover	4.2	0.634	0.865	
Mean temperature	1.2	0.615	0.874	
Mean NDVI	0	0.593	0.869	
Model 2				
Elevation	65.9	0.817	0.769	0.875
Human population distribution	28.0	0.727	0.815	
Land cover	4.3	0.634	0.870	
Max temperature	1.4	0.546	0.876	
Max NDVI	0.5	0.542	0.875	
Model 3				
Elevation	64.3	0.817	0.766	0.862
Human population distribution	27.1	0.727	0.800	
Land cover	4.0	0.634	0.857	
Min NDVI	2.9	0.606	0.875	
Min temperature	1.6	0.575	0.862	
Model 4				
Elevation	66.5	0.812	0.764	0.872
Human population distribution	28	0.732	0.794	
Land cover	5.5	0.640	0.876	

Variants of temperature and NDVI (mean, maximum, minimum) were attempted in different models, but yielded similar minor contributions to the model. AUC, or area under the curve, values approaching 1 indicate the model’s perfect discrimination between suitable and unsuitable areas for the target species, and values near 0.5 indicate performance no better than random. Model 4 was retained for analyses

### ITN ownership and mosquito habitat suitability

**Fig. 7:**
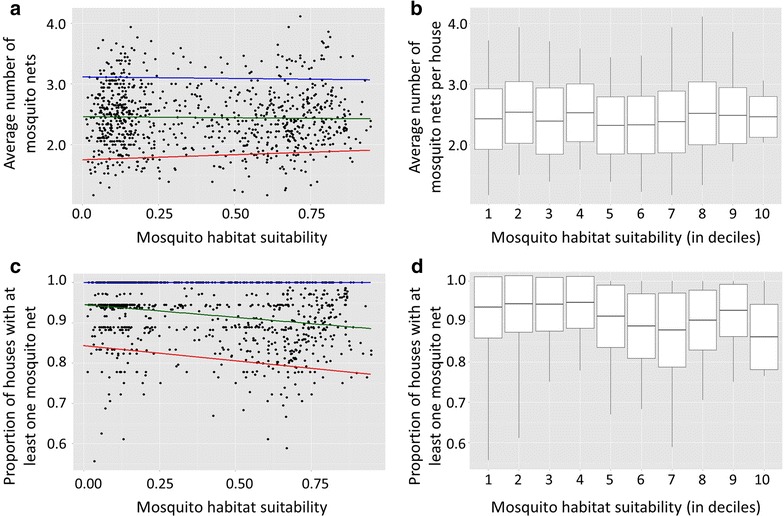
Scatterplots and corresponding *boxplots* of mosquito net use as a function of mosquito habitat suitability. **a** Scatterplot of the average number of mosquito nets per house as a function of relative *Anopheles* habitat suitability with quantile regression and ordinary least squares (OLS) regression lines shown. The 0.9 quantile line is shown in *blue*, the ordinary least squares regression line is shown in *green*, and the 0.1 quantile line is shown in *red*. **b** *Boxplot* of mean and standard deviation of average number of mosquito nets used per house as a function of *Anopheles* relative habitat suitability, where habitat suitability is divided into deciles (e.g. 1 corresponds to lowest 10 %, etc.). *Boxplot whiskers* extend to the maximum and minimum values. **c** Scatterplot of the proportion of houses with mosquito nets as a function of relative *Anopheles* habitat suitability (i.e. houses with ≥1 mosquito net were assigned a value of 1; otherwise, 0), with quantile regression and ordinary least squares (OLS) regression lines shown. The 0.9 quantile line is shown in *blue*, the ordinary least squares regression line is shown in *green*, and the 0.1 quantile line is shown in *red*. **d** *Boxplot* of mean and standard deviation of the proportion of houses with mosquito nets as a function of relative *Anopheles* habitat suitability, where habitat suitability is divided into deciles

The relationship between the average number of mosquito nets owned per house and mosquito habitat suitability was not significant (p > 0.05, Fig. 7a). Average mosquito net ownership per house did not significantly differ between rural (2.485 mosquito nets) and urban (2.491 mosquito nets) clusters (t = 0.13, p = 0.8967). Variance did not significantly differ among deciles of habitat suitability (F = 0.7821, p = 0.6331; Fig. 7b). There is no relationship between mean numbers of ITNs owned per household and predicted habitat suitability among households in areas among highest or lowest mosquito habitat suitability (the top or bottom deciles, or 10 %, of clusters, respectively) (p > 0.05; Fig. 7a).

There is a slight tendency for the proportion of households with at least one mosquito net to decrease toward areas with higher mosquito habitat suitability (R^2^ = 0.051, p < 1 × 10^−6^; Fig. 7c). Overall, 93.41 % of surveyed households had at least one mosquito net (93.51 % in rural households and 93.05 % in urban households). Variance in ITN ownership rates across surveyed households with at least one mosquito net did differ significantly between deciles of habitat suitability (F = 3.0037, p = 0.0015; Fig. 7d). While the 0.9 quantile of the proportion of households with at least one mosquito net showed no significant trend relative to mosquito habitat suitability, the 0.1 quantile of the proportion of households with at least one mosquito net significantly decreased as mosquito habitat suitability increased (β = −0.0753, t = −3.38, p < 0.001; Fig. 7c).

## Discussion

The World Health Organization (WHO) estimates ~50 % of African households had access to ≥1 ITN by 2010 but only ~3 % in 2004 [1]. This study’s analysis of the DHS survey data across Tanzania from 2011 to 2012 suggests more optimistic results, with >93 % of Tanzanian households having one or more nets, meeting the 90 % national target set for 2013 [13]. There is no difference in ITN ownership rates between rural and urban households. However, the effectiveness of ITN distributions varies among communities, many of which have much lower rates of ITN ownership than national averages. Among such communities, the proportion of households with at least one mosquito net is inversely related to mosquito habitat suitability. There is a clear opportunity to improve ITN effectiveness at a national scale by targeting supplemental net distributions in areas where current ownership rates are low but mosquito habitat suitability peaks. The unexpected decrease in ITN presence in some surveyed areas where malaria risk is high likely contributes to persistent, high malaria morbidity and mortality (20,900 deaths in 2013 [59]). Other factors clearly contribute to malaria persistence, including increasing insecticide resistance among *Anopheles* mosquitoes in Tanzania [60–62].

It is imperative that ITNs reach households where mosquito habitat suitability, and thus malaria risk, is highest. Areas with higher mosquito habitat suitability were expected to have the most comprehensive ITN coverage and lowest variance in the number and proportion of households with one or more ITNs, but the opposite trends were observed. In areas with the highest anopheline habitat suitability, proportional ITN presence in households declines below both the national targets for ITN coverage (90 %) and the more modest (≥80 %) international targets (77 % of households have ≥1 ITN; Fig. 7c). In other words, the proportion of households reporting no ITN ownership increases in areas where mosquito habitat suitability is highest. This gap in ITN ownership is consequential from a human health perspective but is not detectable using models describing the response variable’s central tendency. Coarse resolution analyses have revealed similarly uneven mosquito net use relative to malaria endemicity among 23 African countries [14]. If internal transportation networks through which ITNs and supporting programmes are delivered (e.g. road access) caused local variability in ITN ownership rates, those patterns should have shown strong autocorrelation. Causes of variation in ITN ownership and use need investigation. These include beliefs in malaria risk, trust in health workers distributing nets, perceived benefits of the nets, education, number of children under five in the household, and availability and use of other vector control measures [63].

This study’s prediction of mosquito habitat suitability relates strongly to georeferenced malaria cases and to independent distribution models of *Anopheles* mosquito species in Tanzania. The binomial test of omission was repeated with the fixed threshold of 0.1 to test the model against independently-collected data on unique georeferenced records of *Plasmodium falciparum* across Tanzania from the MAP website. This study’s prediction of mosquito habitat suitability corresponded strongly to malaria records from 1985 to 2008 (model omission rate = 0.1246 for 568 geographically-unique *P. falciparum* records) as well as those specifically within the 1999–2003 period used in this study (omission rate = 0.1321 for 107 *P. falciparum* records). Model prediction (based on the fixed threshold of 0.1) was qualitatively consistent with the predicted occurrence maps for *An. gambiae*, *An. arabiensis*, *An. funestus*, and *An. gambiae* s.s. developed independently [64]. However, this study’s model predicted a narrower distribution than Malaria Atlas Project (MAP) predictions, which likely reflects differences in the time period considered and that MAP models are intended for continental applications and have less within-country detail. This study’s predictions for *An. gambiae* s.l. distributions were also constructed using high resolution, regional satellite data and more extensive vector observation data, which may reveal more subtle variation in relative suitability in *Anopheles* habitats. Although this study’s *Anopheles* training records excluded the Lake Victoria region, this study’s model correctly predicted high relative habitat suitability there. This region is a malaria hotspot [65], with malaria steadily spreading to higher altitudes and aggravated by climate variability and poverty [66].

The distribution of anopheline mosquitoes and malaria is strongly influenced by land cover [6, 67] and human population distributions [43, 68] in addition to climatic and topographical factors [43, 69–71]. Elevation also limits *Anopheles* distributions [4, 43, 72] and its omission from ecological niche models can severely degrade both model fit and prediction accuracy [73]. In this study, mosquito habitat suitability increased particularly in cropland/natural land cover, where irrigation and water pooling are common, and in dense shrublands. Land features, such as soil type, are not directly measured in these satellite land cover data but may help explain such findings through differences in soil water holding capacity that can alter mosquito breeding success [67]. Temperature and NDVI explained little unique variation in habitat suitability, though both can relate to mosquito distributions under some circumstances [4, 8, 72, 74]. Temperature shows relatively little spatial variation across Tanzania except along elevation gradients [75], which are more directly measured by the elevation variable. NDVI relates to vegetation greenness, productivity, and moisture availability, which are also strongly related to mosquito reproduction [76]. However, satellite-based land cover measurements provide similar measurements that may more accurately detect the influences of vegetation on mosquito distributions. Furthermore, highly productive vegetation (i.e. areas with high NDVI) can be found in many regions of Tanzania, including at high elevations that are too cold to permit malaria transmission, eroding the capacity for such measures to discriminate between suitable and unsuitable mosquito habitats at this scale of analysis.

While this study’s dataset includes the most comprehensive collection of spatially-unique georeferenced *Anopheles* records for this region to date, systematic, randomized, broad-scale sampling that identifies species in the *An. gambiae* complex would be extremely valuable. Presence-only species observations assembled from an array of sources that differ in sampling effort and geographical focus could bias models toward areas with easier access (e.g. roadsides, proximity to research centres). In addition, lack of species-by-species evaluation for the historical period forced the combination of *Anopheles* species. By combining the occurrence records across the *An. gambiae* s.l. complex, the applicability of model predictions to particular species is limited (e.g. *An. arabiensis* inhabits more arid regions than *An. gambiae* s.s. and *An. funestus,* better tolerating decreasing precipitation and interruptions in rainfall patterns in certain regions of Tanzania [8]), but the relationship with malaria risk remains very strong. Systematic sampling at the species level, including observations of absence, is needed. ITN ownership and subsequent use may differ depending on which *Anopheles* species is locally common (e.g. *An. arabiensis* is more common outdoors [72], rendering it less affected by ITNs than *An. gambiae* s.s. and *An. funestus* [77]). Systematic, repeated sampling of *Anopheles* mosquitoes across Tanzania could significantly improve the capacity to target interventions, and would provide a stronger basis for predicting how distributions of these vectors would change through time [78].

## Conclusions

Insecticide-treated nets have been broadly distributed across Tanzania but ownership is uneven and declines in areas where anopheline mosquitoes are concentrated. The combination of species distribution models, drawing on high-resolution satellite information, and georeferenced mosquito net data can provide useful perspective on the effectiveness of such interventions in areas where georeferenced vector and vector control data exist. These techniques are transferrable to other vector-borne disease systems. With 3.2 billion people at risk of malaria infection worldwide [1], the need to control malaria is critical. Linking ITN ownership rates to validated mosquito species distribution models can help optimize allocation of limited resources, reveal gaps in such intervention programmes, and help minimize malaria cases where risk is greatest.

## Additional files

Additional file 1: R script used for raster brick function. This document contains the annotated code used to perform the raster brick function on the temperature and NDVI data in R. Additional file 2: R script used to create mosquito net buffer zone layer. This document contains the annotated code used to create the layer containing the mosquito net layer, including how the buffer zones were clipped to district boundaries, and averaged for overlapping areas using R. Additional file 3: R script used to calculate average of habitat suitability pixels under each buffer zone. This document contains the annotated code used to calculate the average pixel value under each buffer zone (overlapping areas act as a separate zone with their own averaged value) using R.

## Abbreviations

AIS: AIDS Indicator Survey
DHS: Demographic and Health Surveys
ENM: Ecological niche model
GFATM: Global fund to fight AIDS, tuberculosis and malaria
ITN: Insecticide-treated nets
LLIN: Long-lasting insecticide-treated nets
MAP: Malaria Atlas Project
MODIS: Moderate resolution imaging spectroradiometer
NDVI: Normalized Difference Vegetation Index
SRTM: Shuttle radar topography mission
WHO: World Health Organization
