# Supplementary material for: Where have all the mosquito nets gone? Spatial modelling reveals mosquito net distributions across Tanzania do not target optimal Anopheles mosquito habitats
Source: Malar J. 2015 Aug 19;14:322. doi: 10.1186/s12936-015-0841-x (PMC4539722; doi:10.1186/s12936-015-0841-x)
Supplement: Additional file 1: — R script used for raster brick function. This document contains the annotated code used to perform the raster brick function on the temperature and NDVI data in R. [file 12936_2015_841_MOESM1_ESM.docx]

**Additional file 1: R script used for raster brick function**

#------------------------------------------------------------------------------------------------------------------#

# This document contains the annotated code used to perform the raster brick function on the

# temperature and NDVI data in R (http://www.r-project.org/). Code written by Andrew

# Plowright. Instructions and comments start with a number sign (#) and

# everything written to the end of that line is read by R as a comment, not a command. “Set the

# path” refers to specifying where the data were located in our computer.

#------------------------------------------------------------------------------------------------------------------#

#-----------------For the raster brick function used on the MODIS temperature data-----------------#

##################

# Set up a path to import 8-day temperature layers for 2001:

yearlyPaths2001 <- list.files(path = "C:\\Users\\R\\Documents\\Tp_8day_projected_2001", full.names= TRUE)

##################

# Set imported files as rasters:

yearlyRasters2001 <- lapply(yearlyPaths2001, raster)

##################

# Create raster brick:

tempbrick2001 <- brick(yearlyRasters2001)

##################

# Calculate mean of the raster brick (i.e. the mean of each pixel):

meanTempRaster2001<-calc(tempbrick2001, fun=mean, na.rm = TRUE)

#--------------------For the raster brick function used on the MODIS NDVI data---------------------#

##################

# Set up a path to import monthly NDVI layers for 2001:

NDVIrasters2001 <- list.files(path = "C:\\Users\\R\\Documents\\NDVI_Clipped_2001", full.names= TRUE)

##################

# Tell R that the path you just imported consists of rasters:

rastersNDVI2001 <- lapply(NDVIrasters2001, raster)

##################

# Create the raster brick:

NDVIrasterbrick2001 <- brick(rastersNDVI2001)

##################

# Calculate the mean of the raster brick (i.e. the mean of each pixel):

meanNDVIRaster2001<-calc(NDVIrasterbrick2001, fun=mean, na.rm = TRUE)
