# Supplementary material for: Where have all the mosquito nets gone? Spatial modelling reveals mosquito net distributions across Tanzania do not target optimal Anopheles mosquito habitats
Source: Malar J. 2015 Aug 19;14:322. doi: 10.1186/s12936-015-0841-x (PMC4539722; doi:10.1186/s12936-015-0841-x)
Supplement: Additional file 2: — R script used to create mosquito net buffer zone layer. This document contains the annotated code used to create the layer containing the mosquito net layer, including how the buffer zones were clipped to district boundaries, and averaged for overlapping areas using R. [file 12936_2015_841_MOESM2_ESM.docx]

**Additional file 2: R script used to create mosquito net buffer zone layer**

**#------------------------------------------------------------------------------------------------------------------#**

# This document contains the annotated code used to create the layer containing the mosquito net

# for the mean number of nets used per house (the same methodology can be used again for the

# number of houses with at least one mosquito net). These scripts include how the buffer zones

# were clipped to district boundaries, and averaged for overlapping areas using R (http://www.r-# project.org/). Code written by Andrew Plowright. Instructions and comments start with a

# number sign (#) and everything written to the end of that line is read by R as a comment, not a

# command. “Set the path” refers to specifying where the data were located in our computer.

**#------------------------------------------------------------------------------------------------------------------#**

**#-----------------------------Clip the buffer layer to district boundaries------------------------------#**

##################

# Load relevant libraries:

library(rgdal)

library(rgeos)

##################

# Load data for the layer of 2-km- and 5-km-radius buffer zones, the layer defining the admin2 district boundaries, and the locations of the raw cluster points:

folder <- "C:\\Users\\R\\Documents"

buff <- readOGR(folder, "Combined_buffers")

reg <- readOGR(folder, "Admin2_Districts_2012")

pts <- readOGR(folder, "Clusters")

##################

# Assign each buffer its corresponding district number:

over <- over(pts, reg)

clust.reg <- cbind(pts[["DHSCLUST"]], over[["Dist_numb"]])

colnames(clust.reg) <- c("DHSCLUST", "Dist_numb")

buff[["Dist_numb"]] <- clust.reg[match(buff[["DHSCLUST"]], clust.reg[,"DHSCLUST"]), "Dist_numb"]

##################

# Manually assign district numbers to centroids in the water:

buff[buff[["DHSCLUST"]] == 732, "Dist_numb"] <- 40

buff[buff[["DHSCLUST"]] == 238, "Dist_numb"] <- 2

buff[buff[["DHSCLUST"]] == 430, "Dist_numb"] <- 93

##################

# Clip buffers according to its district:

outList <- vector("list", length = length(buff))

for(i in 1:length(buff)){

if(is.na(buff@data[i, "Dist_numb"])){

outList[[i]] <- buff[i,]@polygons[[1]]

}else{

distNo <- buff@data[i, "Dist_numb"]

distRow <- which(reg[["Dist_numb"]] == distNo)

int <- gIntersection(buff[i,], reg[distRow,])

outpoly <- int@polygons[[1]]

outpoly@ID <- buff[i,]@polygons[[1]]@ID

outList[[i]] <- outpoly}

}

newbuff <- SpatialPolygonsDataFrame(SpatialPolygons(outList), data = buff@data)

##################

# Save output:

writeOGR(newbuff, dsn=“C:\\Users\\R\\Documents”, layer="newbuff", driver = "ESRI Shapefile")

##################

# A separate step then occurs in ArcGIS 10.1 using the “Union” function (within ArcGIS, this

# tool is located here: toolboxes\system toolboxes\analysis tools.tbx\overlay\union). The

# previous output, labelled “newbuff” would be the input in the “Union” function. All other

# options remained in their default settings. The function takes all the sections of overlapping

# buffers and creates separate polygons with them, removing # them from their original buffers. # For this example, we named our output “newbuff2."

**#---------------------------------Average the overlapping buffer areas---------------------------------#**

##################

# Load a shapefile that is the output of the Union geoprocessing function ArcGIS when applied to the buffers.

union <- readOGR("C:\\Users\\R\\Documents", "newbuff2")

# Define the column for each buffer's unique cluster identifier:

col.cluster <- "DHSCLUST"

# Define the column of interest:

col.average <- "Mean_Nets"

# Define name of the output column:

# (The column in the output file that will contain the means of the values from the column of interest)

col.output <- "MeanNets_Res"

# Define output folder and file name:

outFolder <- " C:\\Users\\R\\Documents"

outFile <- "newbuff2_averages"

##################

# Determine which polygons overlap each other

# Create vector to store the row numbers of overlapping polygons in the "union" shapefile:

union.overlaps <- vector("list", length = length(union))

# Cycle through polygons in the "union" shapefile:

for(i in 1:length(union)){

# Extract area for this give polygon:

poly.area <- union[i,]@polygons[[1]]@area

# Which other polygons within the "union" shapefile intersects with this given polygon:

poly.intersects <- which(gIntersects(union[i,], union, byid = T))

# Create vector to store duplicates of this given polygon:

poly.overlaps <- c()

# Of these polygons, check to see which have the same area as the given polygon. If the area

# is identical, then mark these as being duplicates:

for(overlap in poly.intersects){

if(union[overlap,]@polygons[[1]]@area == poly.area){

poly.overlaps <- c(poly.overlaps, overlap)

}

}

# Return this list of duplicates to the "union.duplicates" list

union.overlaps[[i]] <- poly.overlaps

}

##################

# Calculate means of overlapping areas

union.means <- sapply(union.overlaps, function(x){

return(mean(union@data[x, col.average]))

})

##################

# Determine the cluster(s) belonging to each polygon

union.clusters <- lapply(union.overlaps, function(x){

return(union@data[x, col.cluster])

})

# Reformat cluster numbers into a table

max.overlap <- max(sapply(union.clusters,length))

union.clusters.withNA <- lapply(union.clusters, function(x){

c(x, rep(NA, max.overlap - length(x)))

})

union.clusters.table <- do.call(rbind, union.clusters.withNA)

colnames(union.clusters.table) <- paste0("CLUST", 1:max.overlap)

##################

# Create output data

outData <- cbind(union.means, union.clusters.table)

colnames(outData)[1] <- col.output

##################

# Create output polygon

# Determine the row numbers of duplicated polygons:

union.duplicated <- duplicated(union.overlaps)

# Subset "union" shapefile in order to remove duplicated polygons:

union.subset <- union[!union.duplicated,]

# Format output data before attaching to subset:

outData.subset <- data.frame(outData[!union.duplicated,])

row.names(outData.subset) <- row.names(union.subset)

# Attach output data to the subset shapefile:

union.subset@data <- outData.subset

##################

# Write output

writeOGR(union.subset, dsn=” C:\\Users\\R\\Documents”, layer= “union_MeanNets”, driver = "ESRI Shapefile")
