# Supplementary material for: Where have all the mosquito nets gone? Spatial modelling reveals mosquito net distributions across Tanzania do not target optimal Anopheles mosquito habitats
Source: Malar J. 2015 Aug 19;14:322. doi: 10.1186/s12936-015-0841-x (PMC4539722; doi:10.1186/s12936-015-0841-x)
Supplement: Additional file 3: — R script used to calculate average of habitat suitability pixels under each buffer zone. This document contains the annotated code used to calculate the average pixel value under each buffer zone (overlapping areas act as a separate zone with their own averaged value) using R. [file 12936_2015_841_MOESM3_ESM.docx]

**Additional File 3: R script used to calculate average of habitat suitability pixels under each buffer zone**

#------------------------------------------------------------------------------------------------------------------#

# This document contains the annotated code used to calculate the average pixel value under

# each buffer zone (overlapping areas act as a separate zone with their own averaged value)

# using R (http://www.r-project.org/). Code written by Andrew Plowright.

# Instructions and comments start with a number sign (#) and everything written to the end of

# that line is read by R as a comment, not a command. “Set the path” refers to specifying where

# the data were located in our computer.

#------------------------------------------------------------------------------------------------------------------#

##################

# Load relevant libraries:

library(rgdal)

##################

# Set the path to import the habitat suitability layer for all mosquito species for 2001:

suit2001<-raster(“C:\\Users\\R\\Documents\\2001_Anopheles.asc”)

##################

# Set the path to import the bed net polygon data for 2011-2012:

bednetPolygon<-readOGR(dsn=“ C:\\Users\\R\\Documents”, layer="union_MeanNets")

plot(bednetPolygon)

##################

# Compare bed nets and suitability 2001:

suitVals <- extract(suit2001, bednetPolygon)

suitAvg <- sapply(suitVals, mean, na.rm = TRUE)
